# The Human Resources for Health Program in Rwanda: A Response to Recent Commentaries

**DOI:** 10.15171/ijhpm.2019.19

**Published:** 2019-04-17

**Authors:** Corrado Cancedda, Agnes Binagwaho

**Affiliations:** ^1^ Center for Global Health, Perelman School of Medicine, University of Pennsylvania, Philadelphia, PA, USA.; ^2^ Division of Infectious Diseases, Department of Medicine, Perelman School of Medicine, University of Pennsylvania, Philadelphia, PA, USA.; ^3^ Office of the Vice-Chancellor, University of Global Health Equity, Kigali, Rwanda.; ^4^ Department of Global Health and Social Medicine, Harvard Medical School, Boston, MA, USA.; ^5^ Department of Pediatrics, Geisel School of Medicine, Dartmouth College, Hanover, NH, USA.

We are grateful that our article on the first five years of the Human Resources for Health Program (HRH Program) in Rwanda was followed by two very thoughtful and insightful commentaries.^1,2^ The points made and questions raised by the commentaries are very important ones and deserve comprehensive and detailed answers. We hope that the authors of the commentaries and the readers of this journal will find our answers satisfactory and we look forward to additional fruitful and constructive discussions in the future.

## Perceived Prioritization by the HRH Program of Specialized Care Over Primary Care and of Clinical Care Over Public Health

It is important to note that the HRH Program did not represent the totality of the Ministry of Health’s (MoH’s) national strategic plan for the health workforce, but was one (critical) component of such plan.^3^ Independently (but in a complementary and synergistic manner) of the HRH Program, Rwanda continued to train community health workers (CHWs), clinical officers, nutritionists, and other primary care and public health practitioners during the program’s implementation.

CHWs in particular are the backbone of Rwanda’s health system.^4^ Each one of the country’s almost 15 000 villages is served by three CHWs who report to coordinators based in the health centers of reference. CHWs engage in primary care by dispensing artemisinin combination drugs for malaria, amoxicillin for pneumonia, oral rehydration salts and zinc tablets for diarrhea, ready-to-use therapeutic food for acute severe malnutrition and stunting, and oral or injectable contraceptives for birth-control.^5^ CHWs also contribute to public health by promoting healthy behavior within their villages, by sensitizing the public to community-based health insurance, and through monitoring/case detection related to nutrition, maternal and newborn health, HIV/AIDS, and tuberculosis.^5^ An independent evaluation of Rwanda’s national program for CHWs conducted in 2016 concluded that it was highly consistent with national policies and plans and effective in reaching out to remote villages and households.^5^

Because of its investment in primary care and public health, Rwanda was able to achieve all of the health-related millennium development goals and accounts for the impressive improvements observed over the past 25 years across a wide range of health indicators.^6,7^ These indicators include: immunization rates above 90% for diphtheria, tetanus, pertussis, measles, pneumococcus, haemophilus, and poliomyelitis; a drop in fertility rates from 6.3 to 3.9 children per woman; a 91% rate of deliveries performed in the presence of a skilled birth attendance; and a drop in under-five stunting rates from 52% to 39%.^7,8^ As the authors of the two commentaries correctly point out, while these improvements are indeed impressive, more remains to be done, especially for nutrition. For this reason, in early 2019 Rwanda decided to add another 15 000 CHWs to its workforce.

It is important also to note that food security, nutrition and early childhood development are one of the pillars of Rwanda’s National Strategy for Transformation and Prosperity (2017-2024), which calls for a drop of the current under-five stunting rate to 19% by 2024.^9^ To ensure the effective implementation of the strategy and high-level coordination among national and international stakeholders, the Government of Rwanda recently established a National Early Childhood Development Coordination Program that is led by the current Prime Minister.^10^

Because of Rwanda’s previous progress, the HRH Program was designed to be complementary to and synergistic with ongoing primary care and public health interventions and strengthen the continuity between distal (communities and health centers) and proximal (district hospitals and referral hospitals) links in the health service delivery chain.^11,12^

Additionally, not all the training programs initiated or supported by the HRH Program targeted specialists.^11,12^ In fact, only the master of medicine for physicians, the master of sciences for nurses, and the bachelor of science for dental surgeons did (with a projected training output by the summer of 2019 of 305, 209, and 31 respectively, out of a total projected training output for the HRH Program of 4598).^11,12^ The majority of health professionals targeted by the HRH Program were nurses and midwives with an advanced diploma and bachelor’s degree.^11,12^ Many of these nurses and midwives are based within health centers in rural areas, where their work is closely integrated with that of CHWs and they regularly manage respiratory infections, neonatal disorders, diarrheal diseases and malnutrition among children, treat tuberculosis and HIV-AIDS among adults, and provide prenatal care and perform deliveries for pregnant women.

## Programmatic and Financial Sustainability of the HRH Program

While the initial budget for the HRH Program was significant, the program’s programmatic financial sustainability was actively pursued the very beginning.^11,12^ Over time, new graduates from the training programs initiated or supported by the HRH Program were expected to gradually replace the visiting faculty deployed by the US academic institutions.^11,12^ Additionally, the MoH was expected to gradually pick up the costs covered by President’s Emergency Plan for AIDS Relief (PEPFAR) and the Global Fund, as a result of Rwanda’s projected gross domestic product growth and increase in health expenditure.^11,12^ Despite PEPFAR’s early withdrawal, the HRH Program has been working to reconfigure its targets, budget, and timeline in order sustain itself with only the Global Fund’s financial contribution and increased MoH funding.^12^ Additionally, a significant portion of PEPFAR’s financial contribution was not the result of a one-off donation to the HRH Program, but of the reallocation of existing funds from programs and initiatives that were under-delivering.

## The Need for Capacity Strengthening Beyond Purely Clinical and Public Health Competencies and About Retention and Career Development of Newly Trained Health Professionals

The master in global health delivery and the master in hospital administration were designed to ensure that within each level of the health system there would be the required leadership and management skills to support the work of both clinicians and public health practitioners.^11,12^ Additionally, by establishing a management team for the HRH Program within its own Single Project Implementation Unit, the MoH strengthened its own capacity to manage similarly large and complex programs and initiatives in the future. Lastly, with the contribution of the visiting faculty deployed by the US academic institutions, the MoH was able to revise its national policies in areas of critical need such as emergency medicine, non-communicable diseases, palliative care, and pathology.^12^

While the HRH Program was primarily designed to initiate and support training programs at the graduate and post-graduate level within Rwanda’s academic institutions, the visiting faculty deployed by the US academic institutions have helped launch numerous continuing professional development programs targeting practicing physicians, nurses, midwives, oral health professionals, and health administrators.^12^ To further enhance retention of health professionals within the public sector the MoH enforced a 4- to 5-year contract of employment for all new graduates and is looking to improve infrastructure, equipment, and supplies within Rwanda’s health facilities.^12^

## The Need for Curricula to Endorse Task-Shifting and Pedagogic Innovation Such as Inter-professional and Inter-sectoral Training

To optimize health service delivery across all of the country’s health districts, Rwanda has adopted and regulated task-shifting within its health workforce since 2008.^13^ Nurses and physician assistants in health centers regularly perform deliveries and treat patients with HIV/AIDS and tuberculosis, non-communicable diseases, and malnutrition while general practitioners in district hospitals perform cesarean-sections and surgeries. The training and continuing professional development programs available for these types of health professionals (including those initiated or supported by the HRH Program) capture this shift in the tasks performed and prioritize the acquisition of primary care and public health competencies.

Furthermore, the HRH Program fostered significant pedagogic innovations within its training programs such as the adoption of simulation-based training and flipped classroom/case-based approaches, the development of new modules and training materials (some of which are online), and the pursuit of inter-professional training and inter-sectoral training (for example, in in the case of the master in global health delivery).^12^

## The Need to Train the Right Types of Health Professionals to Properly Address Rwanda’s Burden of Disease

The process followed by the MoH to determine the types and number of health professionals that the HRH Program needed to train was pretty linear. First, the MoH developed its national strategic plan for the health workforce with support from the main national health professional bodies.^3^ Second, the MoH determined the ideal staff composition for all health facilities in Rwanda (health centers, district hospitals, and referral hospitals) based on the data provided by the national census (average population served) and health information system (the average burden of disease encountered by the staff within each type of facility). Third, the MoH looked at the types and amounts of health professionals (both clinicians and public health practitioners) that were already being trained or about to be trained at scale as a result of programs and initiatives other than the HRH Program. Fourth, the MoH finalized the scope and targets of the HRH Program based on the gap between the ideal and current staff composition within Rwanda’s health facilities and the health workforce needs which had been left unaddressed by existing or soon to be launched training programs.

## Replicability in Other Countries and Reliance on the Resources and Expertise of Academic Institutions From the Global North Rather Than From the Global South

Rather than by the priorities of donors and implementing partners, the HRH Program was driven primarily by the priorities of Rwanda and by the need to address the health workforce shortage comprehensively and systematically according to the MoH’s national strategic plan for the health workforce. This required significant work upfront (both in terms of time (18 months) and effort) to design the program, engage donors and implementing partners in a harmonized way, and enhance the management administrative infrastructure within the MoH. This also required several operational adjustments to be pursued and corrective actions to be taken during the implementation phase. While the launch and implementation of the HRH Program was a very challenging and complex endeavor, it was ultimately an endeavor worth pursuing not only because of what it sought to achieve, but also because of how it sought to achieve it. We believe that other countries could benefit from adopting a similar approach, with the obvious understanding that each country is unique and what is replicable are not necessarily the specifics of the HRH Program, but rather the guiding principles underpinning them.

In the current system of global health governance there are many quick wins that can be achieved by leveraging the resources and expertise of vertical programs and the self-interest of donors. However, in our experience these wins often risk trading the substance of long-term capacity strengthening and structural change for the positive optics of short-term outputs.The same wins also fail to challenge structural inequities and gaps in accountability that at times affect the interaction of international donors and implementing partners with local governments, academic institutions, and grassroots organizations. Additionally, the overwhelming task of coordinating a multitude of international stakeholders and the pressure (both internal and external) to succeed quickly often prevents governments in the developing world from clearly articulating their vision and strategy through comprehensive and systematic plans. Lastly, the limited control that these governments have over the way foreign funds (often flowing directly from donors to implementing partners) are spent further hinders the execution of such plans. Not unlike other health professional training initiatives implemented over the past ten years (such as the Medical Education Partnership Initiative and the Global Health Service Core Partnership), the HRH Program sought to alter some of these unhealthy dynamics and in the process pushed all the stakeholders involved (both international and local) out of their comfort zone.^11,12,14^

Lastly, the HRH Program relied on the resources and expertise of US academic institutions because academic institutions in sub-Saharan Africa face the same limitations that were faced by academic institutions in Rwanda at the time that the program was launched.Now that Rwanda has strengthened its own capacity to train health professionals, it has begun actively supporting countries in sub-Saharan Africa by hosting their trainees within its academic institutions, deploying its trainers abroad, and participating in medical missions during humanitarian crises in the region.

## Ethical issues

Not applicable.

## Competing interests

Authors declare that they have no competing interests.

## Authors’ contributions

CC and AB both reviewed the points made and questions raised by the two commentaries critiquing the original article on the Rwanda Human Resources for Health Program. CC and AB both articulated and structured the answers to such critique. CC wrote the first draft of this article, and AB, as the more senior author, assisted with the revision and finalization of the draft.

## Authors’ affiliations

^1^Center for Global Health, Perelman School of Medicine, University of Pennsylvania, Philadelphia, PA, USA. ^2^Division of Infectious Diseases, Department of Medicine, Perelman School of Medicine, University of Pennsylvania, Philadelphia, PA, USA. ^3^Office of the Vice-Chancellor, University of Global Health Equity, Kigali, Rwanda. ^4^Department of Global Health and Social Medicine, Harvard Medical School, Boston, MA, USA. ^5^Department of Pediatrics, Geisel School of Medicine, Dartmouth College, Hanover, NH, USA.
